# Impacts of *SAYE PLUS*, an Antimalarial Phytomedicine With Potential Anti-COVID-19, on the Physical, Biological, and Genotoxicity Parameters of Rodents in Short-Term Toxicity Studies

**DOI:** 10.1155/jt/7536185

**Published:** 2025-07-21

**Authors:** Joël Ouédraogo, Sylvain Ilboudo, Geoffroy Gueswindé Ouédraogo, Virginie Dakuyo, Salfo Ouédraogo, Gaétan D. Somda, Jean Claude Romaric Pingdwindé Ouédraogo, Moussa Ouédraogo, Rasmané Semdé, Sylvin Ouédraogo

**Affiliations:** ^1^Department of Traditional Medicine-Pharmacopoeia and Pharmacy, Institute for Research in Health Sciences, National Center for Scientific and Technological Research (MEPHATRA-PH/IRSS/CNRST), Ouagadougou 03 BP 7047, Burkina Faso; ^2^Laboratory for Drug Development, African Center of Excellence for Training, Research and Expertise in Pharmaceutical Sciences, Joseph KI-ZERBO University (LADME/CEA-CFOREM/UJKZ), Ouagadougou 03 BP 7021, Burkina Faso; ^3^International Research Laboratory-Environment, Health, Societies (IRL 3189, ESS) CNRST/CNRS/UCAD/UGB/USTTB, Ouagadougou, Burkina Faso; ^4^Laboratoires PHYTOFLA, Banfora BP 293, Burkina Faso

**Keywords:** Burkina Faso, COVID-19 care, genotoxicity test, lipid peroxidation, SAYE PLUS, sperm quality

## Abstract

In response to the COVID-19 pandemic and following the World Health Organization's call for action, several traditional medicine recipes were used without any scientific prerequisites concerning their safety. The current study investigated several short-term toxicity parameters of SAYE PLUS, an antimalarial phytomedicine used in COVID-19 patients in Burkina Faso. Following the guidelines of the Organisation for Economic Co-operation and Development (OECD), the safety profile of SAYE PLUS was investigated in a battery of tests in rats and mice. In an acute toxicity study, male and female rats received a single oral dose of 2000 mg/kg b.w. of the test substance. For the subacute toxicity test, male and female rats received daily oral doses of 250, 500, and 1000 mg/kg b.w. for 28 days. Acute and subacute toxicity tests were accompanied by food and water intake, body and organ relative weight, and blood chemistry of animals recording. In mutagenicity, sperm quality, and lipid peroxidation tests, mice were orally exposed to daily oral doses of 500, 1000, and 2000 mg/kg for seven days. Single dose of 2000 mg/kg b.w. of SAYE PLUS did not cause rats mortality. The LD_50_ is more than 2000 mg/kg b.w. Daily administration of SAYE PLUS for 28 days did not induce any significant change in the water or food intake and the body or organ relative weights of animals. Furthermore, no significant change was observed in biochemical parameters. In the test conditions, the recipe did not induce an increase of micronucleus or changes in sperm motility and number. However, all tested doses of SAYE PLUS induced a significant increase in MDA levels in mice serum. These results show that SAYE PLUS did not induce negative impacts on studied parameters, but the possible lipidic peroxidation observed must be further investigated for its mechanism and effects.

## 1. Introduction

Throughout human history, people in all countries and cultures have relied on traditional healers, home remedies, and ancestral medicinal knowledge to meet their health and well-being needs. To date, there is a strong public interest in traditional medicine. Almost half the population in many industrialized countries now regularly uses some form of traditional and complementary medicine (T&CM): United States, 42%; Australia, 48%; France, 49%; Canada, 70% [1]. Considerable use of some form of T&CM exists in many other countries, such as Chile (71%), Colombia (40%), and up to 80% in some African countries. Recent data show that the prevalence of traditional medicines recourse in Burkina Faso was 85%, and malaria is the disease against which traditional remedies are most widely used (18.3%) [2].

After the advent of COVID-19, when the world was shaken by what was to be one of the most significant health disasters of all time, no immediate solution was available. Several treatments based on modern antimalarial drugs, such as chloroquine, hydroxychloroquine, azithromycin, pyronaridine, and artemisinin-based combination therapy, have raised hopes [3]. In Burkina Faso, a prominent practitioner of traditional medicine and promoter of herbal recipes has proposed SAYE PLUS to treat COVID-19. The SAYE phytomedicine, composed of *Cassia alata, Cochlospermum planchnii*, and *Phyllanthus amarus*, has been used for decades to treat malaria. Its antimalarial properties have been demonstrated, and some preclinical and clinical data on the efficacy and safety are available [4]. SAYE PLUS, a derivative of SAYE to which *Artemesia annua* has been added, was proposed in 2020 by the same sponsor against COVID-19. It has benefited from a widespread endorsement without any substantial scientific prerequisites.

Moreover, the COVID-19 health crisis has further highlighted the renewed interest of the African population in general, and Burkinabè in particular, in natural remedies for their health care. Natural health products' presumed safety contributes to this high reliance on traditional remedies. However, this presumption of safety has been challenged by several studies around the world, which have reported cases of adverse reactions and other health risks due to the use of traditional medicines [5–7]. In Burkina Faso, Ouoba et al. [2] reported that the frequency of adverse events among traditional medicine recipe users was 14.7%, including mainly gastrointestinal disorders (57.7%), nervous system disorders (15.2%), and skin disorders (7%). It is essential to investigate the safety of recipes used for healthcare. Thus, the present study aimed to investigate the adverse effects of short-term administration of the recipe SAYE PLUS to rodents. Precisely, it was planned (1) to determine the acute and subacute toxicity of the recipe, (2) to assess the ability of the recipe to induce oxidative stress in test animals, (3) to assess the genotoxic potential of the recipe, and (4) to assess the potential impact of the recipe on mouse sperm count and motility.

## 2. Materials and Methods

### 2.1. Drug

The drug consisted of the phytomedicine SAYE PLUS, a powder mixture of four plants: *Artemisia annua, Cassia alata (Senna alata), Cochlospermum planchnii,* and *Phyllanthus amarus*. The drug is supplied in capsules, each containing 125 mg of SAYE PLUS powder. The recommended dosage is 2–3 capsules daily, with a maximum of three daily doses. A random sample of the phytomedicine, Lot 0008/01.2021, was taken from the manufacturer's warehouse. It was ground more finely in the laboratory using an IKA A11 basic Analytical mill and sieved through a 300 μm Retsch GmbH sieve.

### 2.2. Animals

All the study animals were provided by the “Institut de Recherche en Sciences de la Santé” pet Shop. Both healthy male and female Wistar rats (150 and 200 g) and NMRI mice (30 and 37 g) were used. They received during their lifetime water and food ad libitum, maintained under convenient environmental conditions consisting of temperature of 22 ± 3°C, relative humidity of 65 ± 5%, and subjected to cycles of light and darkness of 12/12 h. Female animals were nulliparous and nonpregnants. The study was approved by the Health Research Ethics Committee under Deliberation No. 2021-04-104 on 21/04/2021. Then, all experiments were carried out following ethical guidelines towards animals and on the established principles of Reduction and Refinement, in accordance with the international standards set by the European Union on the protection of animals (CEC Council 86/609).

### 2.3. Acute Oral Toxicity Test

The oral acute toxicity test was performed on female Wistar rats weighing between 150 and 200 g, in accordance with the OECD guideline 423 [8]. Briefly, the animals were randomly allocated into groups of three rats. Prior to the test, they were fasted overnight (from 6 pm on the previous day to 8 am on the day of the test). Test groups received a single oral dose of 2000 mg/kg b.w of fine powder of the drug dissolved in distilled water, while the control group received distilled water. Animals received test substance or distilled water by gavage using a stainless-steel ball-tipped gavage needle. Both control and test animals were observed every 30 min for 2 h after dosing, then twice daily until the 14th day. Observations included changes in skin and fur, eyes and mucous membranes, respiratory, circulatory, autonomic, and central nervous systems, and somatomotor activity and behavior patterns. Special attentions were directed to observations of tremors, convulsions, salivation, diarrhea, lethargy, sleep, and coma.

All rats were euthanized by ketamine *i.p.* administration (1.5 mg/kg b.w. in *i.p.*) at the end of the 14-day observation period, and gross necropsies were performed on all animals. Blood samples were also collected from euthanized animals for serum biochemical analyses. The brain, heart, kidneys, liver, lungs, stomach, spleen, and urinary bladder were removed, rinsed in saline solution(NaCl 0.9%), and weighed.

### 2.4. Subacute Oral Toxicity Test

The test was performed on Wistar rats in accordance with the OECD guideline 407 [9]. Forty Wistar rats were divided into four groups of 10 rats (5 males and 5 females), including one control and three test groups. SAYE PLUS dispersed in water was administered orally through gastric intubation at daily doses of 250, 500, and 1000 mg/kg b.w., and the control group received water alone. The animals were observed at least twice daily throughout the test period for any signs of behavioral changes, toxicity, and mortality. Furthermore, they were weighed before starting the test and every week till the end of the study. Water and feed consumption were recorded (daily for water and weekly for feed) during the 4 weeks of the study. On the evening of the 28th day, the animals were fasted for 12 h, anesthetized by ketamine *i.p.* administration (150 mg/kg b.w.), and blood was collected by cardiac puncture. Organs such as the heart, liver, lungs, kidneys, and spleen were then removed, examined for macroscopic changes, and weighed. The relative weight of each organ was calculated according to the following formula:

(1) $$\begin{array}{c} \text{RWO }\left(\%\right)=\frac{\text{Absolute organ weight }\left(g\right)}{\text{Fasting rat weight }\left(g\right)}\times 100, \end{array}$$

where RWO is the relative weight of organ.

Biochemical parameters such as alanine aminotransferase (ALT), aspartate aminotransferase (AST), total protein, creatinine, and total cholesterol were determined in serum using a MINDRAY BA-88A spectrophotometer.

### 2.5. MDA Determination

Blood of NMRI mice used for sperm morphology and motility essay was collected by cardiac puncture and centrifuged at 3500 rpm for 15 min to recover serum for MDA quantification. MDA levels in serum were determined by measuring thiobarbituric acid reactive substances (TBA) following the slightly modified method of Pal et al. [10]. To 500 μL of serum, 938 μL of 1% orthophosphoric acid was added, centrifuged at 3000 rpm for 10 min, and the supernatant was collected. Then, 315 μL of TBA (0.6%) was added to the supernatant and heated in a water bath at 90°C for 1 h using a Fisher Scientific polyester 20. After cooling (10 min) on ice, absorbance was determined at a wavelength of 532 nm at room temperature using a Shimadzu UV-1800 spectrophotometer. The degree of lipid peroxidation was expressed in MDA (μmol/L) using the molar extinction coefficient for MDA: 155 M^−1^.cm^−1^.

### 2.6. Mutagenicity Test

The mutagenicity test was performed according to the OECD guideline 474 in NMRI mice [11]. Five groups of 10 mice (5 males and 5 females) were formed: a negative control group, a positive control group, and three test groups. Distilled water was administered orally to the negative control, while the positive control received intraperitoneally 80 mg/kg b.w. of potassium bromate (KBrO_3_) in 0.9% NaCl solution [12]. The three test groups received orally, respectively, 500, 1000, or 2000 mg/kg b.w. of SAYE PLUS aqueous suspension. All these substances were administered to the animals once a day for one week. All animals were observed twice daily for morbidity and mortality during the dosing period. At the end of the 7 days of the test, venous blood was collected from the tail vein 24 h after the last administration. Blood smears were taken, dried, fixed with methanol, and stained with 1:10 diluted Giemsa for 20 min. Slides were read under a light microscope at x100 magnification. All slides were coded prior to microscopic examination. The number of normochromatic erythrocytes (NCE) per 100 NCE, the number of polychromatic erythrocytes (PCE) per 100 PCE, and the number of micronucleated normochromatic erythrocytes (MNCE) per 100 MNCE were recorded.

### 2.7. Sperm Quality Test

The effects of the test substance were assessed on NMRI mice sperm morphology and motility in accordance with the OECD 416 guideline [13], slightly modified. Mice were randomly assigned to four groups of 5 animals each: 0 (water), 500, 1000, and 2000 mg/kg b.w. of aqueous suspension of SAYE PLUS. Dosing solutions, prepared once a day, were orally administered daily for 7 days. During the experiment, all animals were examined twice daily for general clinical signs such as hair straightening and alterations in locomotor activity, and mortality. After seven days of treatment, mice were anesthetized with ketamine (1.5 mg/kg b.w. by *i.p.* administration), and the testis and epididymis were isolated [14].

#### 2.7.1. Sperm Motility Determination

Sperm were recovered from the epididymis for sperm motion analysis as previously described [15], with slight modifications. Briefly, the caudate epididymis of mice was nicked with a scalpel blade to harvest sperm in 1 mL of 0.9% NaCl. After homogenization, the sperm suspension was placed in Petri dishes and incubated at 37°C for 10 min to allow the sperm to gain motility. Mass motility was estimated using a phase-contrast microscope at × 100 magnification.

#### 2.7.2. Sperm Concentration Assessment

The cauda sperm suspension used for the motility assays was deposited with a micropipette in hemocytometer chamber (Malassez cells) by capillary action, without air bubbles, and left to stand for 5 min. Counting was then performed using an optical microscope (magnification × 400) on five large squares [12]. The number of sperm per mL was calculated using the formula below:

(2) $$\begin{array}{c} \text{Number of sperm}=\frac{\text{Number of sperm counted}\times \text{dilution factor}}{\text{ Surface area}\left({\text{mm}}^{2}\right)\times \text{Chamber depth}}. \end{array}$$

### 2.8. Data Analysis

Values represented the means ± SEM (standard error of the mean) from control and experimental treatment groups accounting for at least five animals. Statistical analyses were performed with GraphPad PRISM 5.04 (GraphPad Software, Inc., San Diego, CA, USA). Comparisons have used *t*-test or one-way analysis of variance (ANOVA) followed by Dunnett's comparison post-tests. The differences were considered statistically significant for *p* value < 0.05.

## 3. Results

### 3.1. Acute Oral Toxicity

#### 3.1.1. Mortality and General Observations

The results of the acute oral toxicity study showed that no treatment-related toxic symptom or mortality was observed after oral administration of SAYE PLUS at the dose of 2000 mg/kg b.w. Observations in skin and fur, eyes and mucous membranes, respiratory, circulatory, autonomic, and central nervous systems, somatomotor activity, and behavior patterns revealed no treatment-related changes. Similar results were observed in the second step of the test. According to these results, the 50% lethal dose (LD_50_) of the phytomedicine is more than 2000 mg/kg b.w.

#### 3.1.2. Effect of Single Dose of SAYE PLUS on Body Weights in Rats

**Figure 1:**
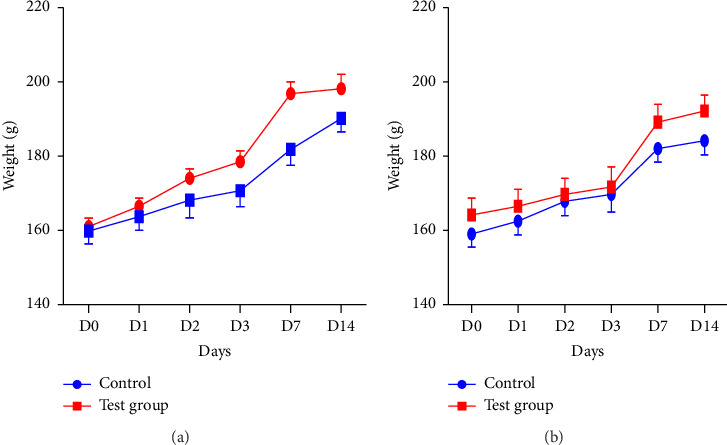
Weight gain of male (a) and female (b) rats over 14 days (D) of observation in the SAYE PLUS acute toxicity study. Comparisons of test group parameters with the control ones used the *t*-test. *n* = 6 per group.

Results show the single oral dose of SAYE PLUS at 2000 mg/kg b.w. did not cause any significant alteration in rats' body weights. Indeed, all rats showed a positive weight trend, as evidenced by the upward trends in the weight curves (Figures 1(a) and 1(b)). Statistical analysis revealed no significant difference (*p* > 0.05) between the test and the control groups.

#### 3.1.3. Effects of Single Dose of SAYE PLUS on the Relative Weights of Specific Organs in Rats

Table 1 depicts the relative organ weight of the heart, liver, lungs, spleen, and kidneys of both treated and control groups rats. Visual observation of these organs revealed that they were not adversely affected by the single oral dose of 2000 mg/kg b.w. Statistical analysis of the relative organ weights of test groups of both sexes compared with controls showed a statistically significant differences between females' lungs and liver relative weights.

#### 3.1.4. Effects of Single Dose of SAYE PLUS on Biochemical Parameters in Rats

The effects of SAYE PLUS single oral dose of 2000 mg/kg b.w. on rats' biochemical parameters are presented in Table 2. The phytomedicine induced statistically significant changes in serum creatinine in both sexes of rats compared to the control. Furthermore, sex-specific changes were noted with serum total cholesterol, electrolytes (PO_4_^2−^, Cl^−^, Na+, K^+^), and AST.

### 3.2. Subacute Toxicity Results

#### 3.2.1. Mortality and General Observations

During the subacute study period (28 days), no mortality was recorded after rats dosing with SAYE PLUS at the daily oral doses of 250, 500, and 1000 mg/kg b.w. No signs of toxicity were observed in the phytomedicine-treated group compared to the control ones.

#### 3.2.2. Effects of Subacute Administration of SAYE PLUS on Food and Water Intakes in Rats

Average water and feed intake by SAYE PLUS-treated and control rats are recorded in Tables 3 and 4, respectively. When compared to the control, the single daily administration of SAYE PLUS at the doses of 250, 500, and 1000 mg/kg b.w. for 28 days caused no statistically significant changes (*p* > 0.05) in food and water intakes.

#### 3.2.3. Effects of Subacute Administration of SAYE PLUS on Body Weights in Rats

The daily oral administration of SAYE PLUS phytomedicine (250, 500, and 1000 mg/kg b.w.) for 28 days did not produce any significant change (*p* > 0.05) in rat body weight compared to the control in both sexes (Table 5).

#### 3.2.4. Effects of Subacute Administration of SAYE PLUS on Relative Specific Organ Weights in Rats

Table 6 displays the relative organ weight of the heart, liver, lungs, spleen, and kidneys of both treated and control groups of rats. These organs' observations revealed no related-treatment visual changes were noted at repeated oral doses of 250, 500, and 1000 mg/kg b.w. throughout the study period (28 days). Furthermore, statistical analysis showed no difference between the relative organ weights of treated and control rat groups (*p* > 0.05).

#### 3.2.5. Effects of Subacute Administration of SAYE PLUS on Biochemical Parameters in Rats

Table 7 presents the results of the subacute oral administration of SAYE PLUS effects on rats' biochemical parameters. Up to a dose of 1000 mg/kg b.w./day, no statistically significant disturbance (*p* > 0.05) was recorded in the levels of rats' biochemical parameters measured at the end of the 28-day study.

### 3.3. Lipid Peroxidation Results

Table 8 shows an increase in MDA concentration in treated batches compared with controls. A statistically significant increase of MDA was observed at all tested doses in both male and female mice.

### 3.4. Mutagenicity Results

**Figure 2:**
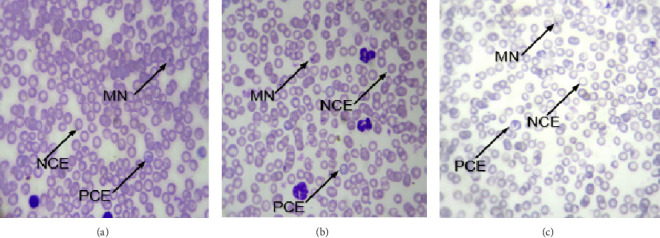
Microscopic observation showing micronucleated erythrocytes, monochromatic and polychromatic erythrocytes (× 100 optics) on negative control mice (a), positive control mice (b), and mice treated with SAYE PLUS with a dose of 2000 mg/kg b.w./day (c). MN: micronuclei. NCE: normochromatic erythrocyte. PCE: polychromatic erythrocyte.

Figure 2 illustrates the micronuclei (MN) found on the peripheral blood smear of mice from the controls and treated groups in microscopic fields. Daily administration of SAYE PLUS for 7 days did not cause any genotoxicity in the experimental groups relative to the control.

**Figure 3:**
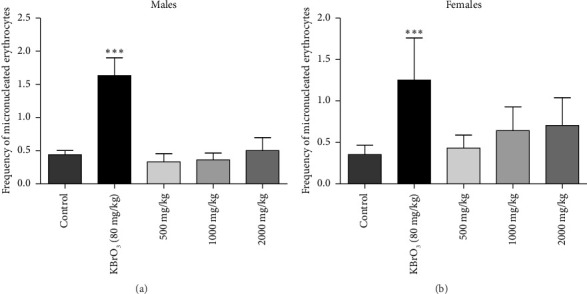
Frequency of micronucleated erythrocytes in the peripheral blood of male (a) and female (b) mice.

Daily administration of SAYE PLUS at dose of 500, 1000 and 2000 mg/kg b.w. for 7 days did not cause any significant (*p* > 0.05) change in number of NCE, PCE, and MNCE in treated mice compared to control ones (Figures 3(a) and 3(b)). The test substance did not induce an increase in MN, and there was no significant difference in the frequency of MNCE between the lowest dose (500 mg/kg b.w.), the highest dose (2000 mg/kg b.w.), and the negative control. However, in mice treated with the positive control potassium bromide (KBrO_3_), there was a significant increase (*p* < 0.05) in the frequency of MNCE in both males and females, compared with the negative control batch.

The number of PCE per 1000 erythrocytes and the PCE/NCE ratio are presented in Table 9. The results show a significant increase in the frequency of MN in the erythrocytes of mice treated with KBrO_3_ (positive control) compared with negative controls. On the other hand, no significant differences were observed in mice treated at different doses of SAYE PLUS compared to negative controls. There was also no significant difference in the number of PCE and the PCE/NCE ratio in treated mice compared with control ones.

### 3.5. Sperm Quality

#### 3.5.1. Mobility

**Figure 4:**
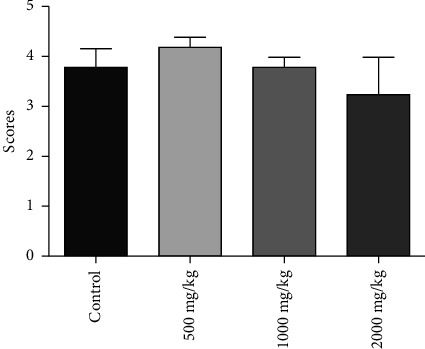
Effect of SAYE PLUS administration for 7 days on sperm mass mobility in male mice.

Daily administration of SAYE PLUS for 7 days did not cause any significant alteration (*p* > 0.05) of the sperm mass mobility in the experimental groups relative to the control (Figure 4). The results revealed that the sperm mass mobility of mice was not adversely affected throughout the treatment period.

#### 3.5.2. Sperm Count

**Figure 5:**
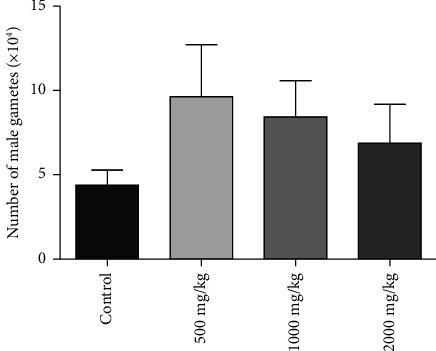
Effect of SAYE PLUS administration for 7 days on sperm count mobility in male mice.

Figure 5 shows the sperm count of mice after the 7-day treatment with SAYE PLUS. The daily oral administration of aqueous suspension of SAYE PLUS at doses of 500, 1000 and 2000 mg/kg b.w. did not show significant change (*p* > 0.05) in sperm count compared to the control mice.

## 4. Discussion

On March 2020, WHO declared Coronavirus disease 2019 (COVID-19) a global pandemic, its first such designation since declaring H1N1 influenza a pandemic in 2009. In January 2020, it embarked on an ambitious global “mega trial” called SOLIDARITY to assess the therapeutic potential of existing drugs against COVID-19 [16]. At the same time, in a call to action, WHO invited traditional medicine practitioners and research centers to get involved in the search of COVID-19 remedies. SAYE PLUS, derived from a recipe traditionally used against malaria, was proposed in Burkina Faso. The initiative was spurred on by information from them indicating an interest in antimalarial treatments against COVID-19. Indeed, verifiable treatment with repurposed antimalarial drugs such as chloroquine, hydroxychloroquine, azithromycin, pyronaridine, and Artemisinin-based combination therapy was going on [17]. The health emergency at the time led to using SAYE PLUS without sufficient prior assessment of its safety. Hence, there is a need to assess the recipe's toxicological profile through short-term toxicity tests in which several parameters were determined.

In the current study, several parameters were assessed after in vivo acute and subacute SAYE PLUS powder suspension administration to rodents. As an essential criterion in toxicological assessment, mortality was recorded during the tests. The results show that no death was recorded in both acute and subacute toxicity test groups. In the acute toxicity study, no death or toxicity-associated symptoms were observed in rat treated with SAYE PLUS at the dose of 2000 mg/kg b.w. With no animals dying, the LD_50_ of the recipe for both sexes of rats is more than 2000 mg/kg b.w. according to OECD 423 guidelines [8]. According to the Globally Harmonized System (GHS) of Classification and Labelling of Chemicals, as SAYE PLUS, the substances with oral LD_50_ values more than 2000 mg/kg b.w. are considered Slightly hazardous [18]. Acute oral toxicity studies carried out with the constituent plants of SAYE PLUS showed that each of them was safe and well tolerated by test rats, even at very high doses [19–22].

Several physical parameters are also used to assess the toxicity of test substances. Thus, changes in body weight and relative organ weights are reliable indicators of the harmful effects of substances on test animals. In the present study, administration of SAYE PLUS, either as a single dose or in repeated doses over 28 consecutive days, did not affect the animal's body weight. Interestingly, treated rats had no significant changes in food or water consumption throughout the 28-day study. The recipe had no impact on feed consumption or weight development.

In the acute toxicity study, a statistically significant rise was noted in female rats' lung and liver relative weight. It is well established that the weights of the organs are markers of pathological and physiological wellness status of animals [23]. Thus, changes in organ weights are hallmarks of toxicity in experimental animals, determined by toxicity tests [24]. Some authors interpret the increase in relative organ weight as a sign of congestion and/or inflammation [25]. As underlined by Sellers et al., organ-detectable weight changes in and of themselves may not necessarily be treatment-related or adverse. Organ weight changes without macroscopic or microscopic correlation should be interpreted with caution [26]. These results highlight the need for histopathological analyses in future studies to better understand the origin of changes in relative organ weights. Likewise, a statistically significant decrease was observed in creatinine, AST, total cholesterol, and Cl^−^ ions in male rats. Similarly, PO_4_^2−^, Na^+^, and K^+^ ions in SAYE PLUS-treated male rats showed a statistically significant increase (Table 2). As previously reported, alteration in the levels of some electrolytes, such as Na^+^, K^+^, and Cl^−^, can be a sign of renal injury [27]. One can speculate that SAYE PLUS at a single oral dose of 2000 mg/kg b.w. would be at the origin of renal impairment. At the same time, a statistically significant drop in creatinine levels was observed in both male and female rats. However, it would be difficult to link a drop in creatinine levels to possible kidney problems. Indeed, the main factors associated with low serum creatinine levels are low muscle mass, malnutrition, advanced liver disease, fluid overload, and augmented renal clearance [28]. For a better understanding of the role of SAYE PLUS in a possible nephrotoxic effect, a study evaluating biochemical parameters including the basic ones [29] as creatinine clearance, urea clearance, glomerular filtration rates (GFRs), and complete serum and urine electrolytes would be done.

Regarding hepatic parameters, the results show a drop in male rat ASAT and an increase in female rat relative liver weight. Surprisingly, the results of the subacute toxicity study did not confirm any of the acute toxicity test effects. It is assumed that these may be transient disturbances that only occur at very high doses. Serum AST, ALT, and total protein concentrations, clinical biomarkers commonly used to assess liver condition [30], were not significantly affected.

Overall, the acute toxicity results for SAYE PLUS show disturbances in parameters that may be precursors of organ damage. It should be remembered, however, that the recommended dosage of SAYE PLUS is 2–3 capsules per day, with a maximum of three daily doses (1125 mg/d). For a 60-kg patient, the maximum recommended dose is equivalent to 18.75 mg/kg/day, more than a hundred (106) times the dose tested in acute toxicity (2000 mg/kg/d). It is doubtful that such effects would be observed under real-life conditions. Further studies are essential to confirm and elucidate the mechanisms involved in such potential lesions through histopathological analysis and repeated-dose toxicity testing. It is therefore important to emphasize the need to use herbal medicinal recipes with caution, and respect recommended doses.

To further understand the possible mechanisms involved in the toxicity of the recipe at very high doses, we set out to measure the level of oxidative stress in laboratory animals. The recipe was administered to male and female mice at daily doses of 500, 1000, and 2000 mg/kg for one week. Results showed that by day 7, all doses of the recipe induced a statistically significant increase in lipid peroxidation levels. Oxidative stress is one of the common mechanisms by which substances exert their toxicity. MDA is the principal and most extensively studied compound derived from lipid peroxidation, known to possess mutagenic and toxic effects [31]. The significant increase in MDA levels results from increased reactive oxygen species (ROS) attacking polyunsaturated fatty acids in the cell membrane, inducing lipid peroxidation [32]. At low doses, free radicals have a regulatory action involved in many physiological processes, notably by activating various cell signaling pathways [33]. Excessive free radicals have a deleterious effect on cells, oxidizing DNA, proteins, and lipids and leading to cell death [34]. Damage caused by free radicals can involve disturbances to the reproductive system, genotoxicity, and many other effects. As all doses tested induced significant lipid peroxidation, one can speculate a potential risk of oxidative damage to organs, especially in patients with chronic use of the drug. Some plant component of SAYE PLUS as Artemisinin is known to have oxidative stress properties [35]. Studies at relatively lower doses should be carried out to establish the dose–response relationship and to determine the threshold dose at which the pro-oxidative effect occurs.

In the present study, the genotoxic potential of SAYE PLUS was assessed using the mouse micronucleus test. Among the in vivo genotoxicity tests, the micronucleus test is one of the most commonly used to evaluate the level of DNA damage [36]. It is well established that MN can occur in almost all dividing cells, but mouse bone marrow is usually the tissue used for the micronucleus test, and any agent which induces chromosomal aberrations can also produce MN [37, 38]. The micronucleus test is part of the battery of tests performed in the current study to assess the preclinical safety profile of SAYE PLUS. Results showed that repeated recipe administration for seven consecutive days failed to induce micronucleus formation in mice (Figures 2 and 3). The potassium bromate (KBrO_3_), known to induce MN in vivo in polychromatic mouse erythrocytes during hematopoiesis [39], caused a significant increase in the frequency of MN in the erythrocytes of mice treated with KBrO_3_ (positive control) in both males and females compared with negative controls (*p* < 0.05). Absence of SAYE PLUS treatment–related genotoxicity is consistent with the lack of increase of the number of immature erythrocytes (PCE) relative to mature erythrocytes (NCE). According to Vilar et al., when a toxic agent affects the normal proliferation of bone marrow cells, the number of immature erythrocytes (PCE) decreases relative to mature erythrocytes (NCE) [40]. Therefore, SAYE PLUS does not affect the normal proliferation of bone marrow cells. Based on the results, it is concluded that test article shows no genotoxicity effects up to the daily oral dose of 2000 mg/kg b.w. for seven days on both male and female mice.

The potential effect of the phytomedicine on some semen parameters was evaluated in this study. The results showed that after one week's repeated exposure to the phytomedicine at daily oral doses of 500, 1000, and 2000 mg/kg b.w., no change was observed in sperm count and motility. They suggest that SAYE PLUS does not induce sperm toxicity under the conditions of the study. More importantly, a nonsignificant increase in sperm count was observed (Figure 5). The maximum increase is observed with the dose of 500 mg/kg b.w. An increase in sperm count may be due to an induction of spermatogenesis. It is shown that *Artemisia annua*, one of SAYE PLUS components, improves spermatogenesis in rats [41]. Abu showed that hydroethanol extracts of *Cochlospermum planchonii*, another SAYE PLUS component, improved spermatogenesis in male rats by significantly increasing sperm motility and count [42]. Another mechanism, such as sperm fluid reduction, could be involved in the increase in sperm count. Indeed, among the multiple functions of the epididymis is fluid resorption, leading to a progressive concentration of sperm in the lumen [43]. The process of fluid resorption in the epididymis has also been shown to be under the control of testicular androgens and adrenergic neurotransmitters [14, 44]. Dharmiyanti and Pangkahila showed that when administered orally, ethanolic extract of *Cassia alata* leaves at 10 mg/kg b.w. resulted in a nonsignificant increase in testosterone levels in male rats [45]. It is, therefore, possible that, at a significantly higher dose (500–2000 mg/kg b.w.), the test article may have a more significant effect on testosterone, with an impact on sperm fluid regulation at the epididymis. Under the conditions of this study, the results clearly show that there are no adverse effects on sperm quality. It should be noted, however, that it is important to carry out a full study with adequate exposure time to detect the effects of SAYE PLUS on spermatogenesis. A specific reprotoxicity study should be carried out to elucidate the trend observed in the results of the present study.

## 5. Conclusion

In the present study, a set of in vivo tests were carried out to assess the toxicity of the phytomedicine SAYE PLUS. The results showed that administration of the test article at a single high dose or in repeated doses did not cause mortality in Wistar rats. However, at the single dose of 2000 mg/kg b.w., a disturbance of biochemical parameters was observed. This disturbance was not observed in the subacute toxicity study. The study of potential genotoxic effects and effects on sperm parameters showed that SAYE PLUS is tolerated. However, an increase in MDA levels was observed at all doses tested in mice. Although they occur at doses much higher than those recommended for the use of the phytomedicine, further investigations are needed to better elucidate them and their mechanisms.

## Figures and Tables

**Table 1:** Effects of single oral dose of SAYE PLUS (2000 mg/kg b.w.) on the relative weights of specific organs in rats.

Organs	Sex	Doses (mg/kg b.w)	
		0	2000
Heart	M	0.36 ± 0.07	0.39 ± 0.03
	F	0.40 ± 0.17	0.48 ± 0.22

Lungs	M	0.49 ± 0.11	0.59 ± 0.04
	F	0.53 ± 0.09	0.69 ± 0.12^∗^

Liver	M	2.80 ± 1.06	3.01 ± 0.14
	F	2.96 ± 0.27	3.64 ± 0.61^∗^

Kidneys	M	0.71 ± 0.07	0.65 ± 0.04
	F	0.68 ± 0.09	0.69 ± 0.05

Spleen	M	0.19 ± 0.03	0.22 ± 0.03
	F	0.27 ± 0.04	0.25 ± 0.03

*Note:* Values are expressed as mean ± standard deviation; *n* = 6 (males (M) or females (F)). A statistically significant difference between treatment and control groups is set to *p* < 0.05.

^∗^ *p* < 0.05.

**Table 2:** Effects of oral single dose of SAYE PLUS (2000 mg/kg b.w.) on biochemical parameters in rats.

Parameters	Sex	Doses (mg/kg b.w)	
		0	2000
CREAT (μmol/L)	M	62.83 ± 6.96	52.78 ± 3.84^∗^
	F	64.40 ± 3.86	57.28 ± 4.28

Total cholesterol (mmol/L)	M	1.51 ± 0.53	0.89 ± 0.31^∗^
	F	1.32 ± 0.40	0.99 ± 0.31

Total protein (g/L)	M	45.17 ± 10.40	44.08 ± 8.88
	F	49.18 ± 5.84	44.57 ± 9.59

Ca^2+^ (mmol/L)	M	2.23 ± 0.31	2.50 ± 0.21
	F	2.56 ± 0.16	2.66 ± 0.32

PO_4_^2−^ (mmol/L)	M	2.06 ± 0.30	2.99 ± 0.37^∗∗∗^
	F	2.29 ± 0.20	2.81 ± 0.62

Cl^−^ (mmol/L)	M	106.80 ± 2.40	100.30 ± 4.63^∗^
	F	95.17 ± 12.19	95.00 ± 2.19

Na^+^ (mmol/L)	M	134.00 ± 3.69	144.20 ± 6.73^∗^
	F	138.80 ± 3.54	141.80 ± 1.72

K^+^ (mmol/L)	M	4.53 ± 0.92	5.61 ± 0.63^∗^
	F	4.97 ± 0.26	6.30 ± 2.14

AST (UI/L)	M	151.50 ± 24.95	100.20 ± 12.98^∗∗^
	F	112.00 ± 45.80	111.30 ± 24.78

ALT (UI/L)	M	39.67 ± 6.89	35.17 ± 8.93
	F	36.17 ± 19.24	38.50 ± 18.64

*Note:* CREAT: creatinine; AST: aspartate aminotransferase; ALT: alanine aminotransferase. Values are expressed as mean ± standard deviation; *n* = 6 (males (M) or females (F)). Statistically significant difference between treatment and control groups is set to *p* < 0.05.

^∗^ *p* < 0.05.

^∗∗^ *p* < 0.01.

^∗∗∗^ *p* < 0.001.

**Table 3:** Effects of SAYE PLUS on rats' water intake (mL/rat/d) during the subacute toxicity study.

Dates	Sex	Doses (mg/kg b.w./day)			
		0	250	500	1000
W1	M	45.00 ± 5.77	36.86 ± 4.56	37.14 ± 4.63	35.86 ± 5.05
	F	32.00 ± 4.50	45.71 ± 8.86	36.29 ± 4.54	37.71 ± 4.07

W2	M	45.00 ± 4.08	36.43 ± 2.44	42.71 ± 2.56	42.43 ± 2.50
	F	35.00 ± 4.08	47.29 ± 5.06	46.43 ± 6.267	42.29 ± 4.68

W3	M	44.00 ± 4.12	40.00 ± 0.00	42.29 ± 2.36	42.86 ± 2.67
	F	36.43 ± 2.44	47.43 ± 4.43	45.00 ± 4.08	41.29 ± 3.63

W4	M	49.20 ± 3.19	43.57 ± 3.78	45.71 ± 4.49	47.00 ± 4.97
	F	35.29 ± 2.36	47.14 ± 10.35	48.57 ± 6.90	44.71 ± 7.57

*Note:* Values are expressed as mean ± standard deviation; *n* = 5 (males (M) or females (F)). No statistically significant difference between SAYE PLUS–treated groups and control one (*p* > 0.05). W1 (Week 1), W2 (Week 2), W3 (Week 3), and W4 (Week 4).

**Table 4:** Effects of SAYE PLUS on rats' food intakes (g/rat/d) during the subacute toxicity study.

Dates	Sex	Doses (mg/kg b.w./day)			
		0	250	500	1000
W1	M	23.54	19.71	19.14	19.29
	F	23.94	19.14	19.26	20.80

W2	M	27.03	19.49	19.86	22.09
	F	22.31	17.60	18.80	21.23

W3	M	27.94	21.40	22.40	24.46
	F	27.66	17.71	19.34	22.06

W4	M	28.09	25.71	27.60	29.20
	F	28.54	25.20	24.23	25.49

*Note:* Values are expressed as mean, *n* = 5 (males (M) or females (F)). W1 (Week 1), W2 (Week 2), W3 (Week 3), and W4 (Week 4).

**Table 5:** Effects of subacute administration of SAYE PLUS on the body weights in rats.

Group	Body weight (g)				
	Week 0	Week 1	Week 2	Week 3	Week 4
*Male*					
Control	161.75 ± 10.90	187.30 ± 9.53	198.80 ± 8.99	217.50 ± 10.38	230.00 ± 8.48
250 mg/kg/d	163.00 ± 4.24	173.30 ± 4.57	188.30 ± 4.99	209.50 ± 8.58	221.80 ± 8.26
500 mg/kg/d	165.00 ± 8.12	197.50 ± 10.08	203.30 ± 6.02	220.30 ± 8.50	231.00 ± 5.48
1000 mg/kg/d	164.00 ± 8.21	176.50 ± 7.94	188.00 ± 10.10	209.50 ± 4.36	222.30 ± 2.87

*Female*					
Control	146.00 ± 9.19	163.80 ± 10.71	172.60 ± 6.19	177.80 ± 6.83	185.80 ± 4.71
250 mg/kg/d	146.00 ± 9.19	163.40 ± 7.37	165.40 ± 7.76	174.00 ± 6.00	177.80 ± 6.94
500 mg/kg/d	145.80 ± 9.36	163.80 ± 8.58	164.60 ± 6.80	173.40 ± 6.50	176.80 ± 8.55
1000 mg/kg/d	145.40 ± 9.42	161.60 ± 10.29	167.20 ± 9.78	176.00 ± 10.39	178.60 ± 9.07

*Note:* Values are expressed as mean ± standard deviation; *n* = 5 (males (M) or females (F)). Statistically significant difference between treatment and control groups is set to *p* < 0.05.

**Table 6:** Effects of subacute administration of SAYE PLUS on relative organ weights in rats.

Organs	Sex	Doses (mg/kg b.w./day)			
		0	250	500	1000
Heart	M	0.34 ± 0.03	0.28 ± 0.16	0.34 ± 0.04	0.33 ± 0.04
	F	0.36 ± 0.02	0.41 ± 0.03	0.34 ± 0.03	0.33 ± 0.05

Lungs	M	0.52 ± 0.08	0.35 ± 0.09	0.59 ± 0.06	0.55 ± 0.14
	F	0.63 ± 0.05	0.63 ± 0.06	0.55 ± 0.02	0.55 ± 0.07

Liver	M	2.88 ± 0.35	2.92 ± 0.43	3.67 ± 0.47	3.21 ± 0.48
	F	4.07 ± 0.51	3.67 ± 0.14	3.15 ± 0.43	3.27 ± 0.45

Kidney	M	0.74 ± 0.62	0.69 ± 0.54	0.59 ± 0.68	0.67 ± 0.64
	F	0.08 ± 0.07	0.06 ± 0.07	0.09 ± 0.05	0.09 ± 0.09

Rate	M	0.25 ± 0.06	0.22 ± 0.07	0.26 ± 0.05	0.25 ± 0.05
	F	0.04 ± 0.02	0.04 ± 0.02	0.09 ± 0.04	0.07 ± 0.03

*Note:* Values are expressed as mean ± standard deviation; *n* = 5 (males (M) or females (F)). There was no significant difference between the SAYE PLUS treatment and control groups (*p* > 0.05).

**Table 7:** Effects of subacute administration of SAYE PLUS on biochemical parameters in rats.

Parameters	Sex	Doses (mg/kg b.w./day)			
		0	250	500	1000
CREAT (μmol/L)	M	80.36 ± 12.19	91.78 ± 6.95	89.84 ± 14.88	79.26 ± 11.89
	F	100.22 ± 12.64	86.58 ± 13.71	95.98 ± 3.23	90.62 ± 7.88

Total cholesterol (mmol/L)	M	1.72 ± 0.45	1.67 ± 0.21	1.69 ± 0.43	1.61 ± 0.08
	F	1.85 ± 0.44	1.57 ± 0.10	1.87 ± 0.51	1.69 ± 0.35

Total protein (g/L)	M	63.02 ± 4.69	64.36 ± 3.92	63.22 ± 3.49	60.04 ± 2.42
	F	64.04 ± 6.01	63.66 ± 3.62	65.92 ± 4.14	65.26 ± 5.52

AST (UI/L)	M	145.70 ± 25.20	145.14 ± 26.25	132.34 ± 7.62	122.88 ± 11.90
	F	112.54 ± 24.58	117.7 ± 14.54	111.58 ± 18.97	123.16 ± 21.53

ALT (UI/L)	M	53.80 ± 7.50	55.88 ± 14.86	70.44 ± 24.15	53.96 ± 7.93
	F	47.92 ± 9.07	54.42 ± 8.99	43.76 ± 7.04	51.56 ± 7.33

Cl^−^ (mmol/L)	M	100.96 ± 0.67	101.84 ± 1.79	101.62 ± 0.97	103.18 ± 0.33
	F	101.6 ± 1.71	101.82 ± 1.02	101.7 ± 2.35	102.56 ± 2.21

PO_4_^2−^ (mmol/L)	M	3.21 ± 0.17	3.04 ± 0.52	3.97 ± 0.88	3.27 ± 0.47
	F	3.27 ± 0.61	3.32 ± 0.53	2.92 ± 0.52	2.94 ± 0.17

Ca^2+^ (mmol/L)	M	3.25 ± 0.07	3.26 ± 0.17	3.40 ± 0.26	3.40 ± 0.34
	F	3.53 ± 0.15	3.3 ± 0.13	3.38 ± 0.15	3.76 ± 0.26

K^+^ (mmol/L)	M	4.85 ± 1.04	4.05 ± 1.17	6.36 ± 3.21	4.85 ± 1.26
	F	6.55 ± 1.61	4.86 ± 1.17	4.10 ± 0.83	5.55 ± 1.34

*Note:* Values are expressed as mean ± standard deviation; *n* = 5 (males (M) or females (F)). No significant difference between SAYE PLUS treatment groups and control group (*p* > 0.05).

**Table 8:** Effect of administration of SAYE PLUS on serum MDA concentrations (μmol/L) in mice.

Sex	Doses (mg/kg b.w./day)			
	0	500	1000	2000
M	26.54 ± 3.95	35.80 ± 5.98^∗^	42.07 ± 1.76^∗∗∗^	41.00 ± 4.50^∗∗∗^
F	26.09 ± 4.30	54.80 ± 5.45^∗∗∗^	55.69 ± 5.55^∗∗∗^	55.85 ± 1.18^∗∗∗^

*Note:* Values are expressed as mean ± standard deviation; *n* = 5 (males (M) or females (F)).

^∗^ *p* < 0.05.

^∗∗∗^ *p* < 0.001.

**Table 9:** Effect of administration of SAYE PLUS on the number of polychromatic erythrocytes per 1000 erythrocytes in mice.

Mutagenicity parameters	Sex	KBrO_3_	Control	Doses (mg/kg b.w.)		
				500	1000	2000
PCE	M	117.62 ± 49.90^∗∗∗^	20.16 ± 2.52	18.30 ± 2.14	17.00 ± 0.71	13.00 ± 1.47
	F	66.98 ± 7.85^∗∗∗^	21.33 ± 1.53	15.60 ± 2.43	13.20 ± 2.41	14.13 ± 0.85

PCE/NCE (%)	M	12.23 ± 2.69^∗∗^	3.806 ± 2.41	1.800 ± 0.23	1.68 ± 0.08	1.325 ± 0.13
	F	6.45 ± 1.96^∗∗∗^	2.69 ± 0.67	1.58 ± 0.25	2.28 ± 2.09	1.40 ± 0.08

*Note:* Values are expressed as mean ± standard deviation; *n* = 5 (males (M) or females (F)).

^∗∗^ *p* < 0.01.

^∗∗∗^ *p* < 0.001.

## Data Availability

The data that support the findings of this study are available from the corresponding author upon reasonable request.
